# How the economic recession has changed the likelihood of reporting poor self-rated health in Spain

**DOI:** 10.1186/s12939-015-0285-5

**Published:** 2015-12-18

**Authors:** Elena Arroyo, Gemma Renart, Marc Saez

**Affiliations:** Research Group on Statistics, Econometrics and Health (GRECS), University of Girona, Spain, Carrer de la Universitat de Girona 10, Campus Montilivi, 17071 Girona, Spain; CIBER of Epidemiology and Public Health (CIBERESP), Madrid, Spain

**Keywords:** Self-rated health, Chronic conditions, Health econometrics, Economic downturn, Health surveys

## Abstract

**Background:**

Between 2006 and 2011 self-rated health (SRH) (the subjective report of an individual’s health status) actually improved in Spain despite its being in the grips of a serious economic recession. This study examines whether the likelihood of reporting poor health has changed because of the global financial crisis. It also attempts to estimate the differences between SRH and other self-perceived measures of health among groups before and during the current economic crisis in Spain.

**Methods:**

Cross-sectional population-based surveys were conducted in Spain (ENSE 2006 and ENSE 2011) and in Catalonia (ESCA 2006 and ESCA 2011) in 2006 and again in 2011. In this research work we have used random effects logistic models (dependent variable SRH 1 Poor, 0 Good) and exact matching and propensity score-matching.

**Results:**

The results of the ENSE explanatory variables are the same in both 2006 and 2011. In other words, all diseases negatively affect SRH, whereas alcohol habits positively affect SRH and obesity is the only disease unrelated to SRH. ESCA explanatory variables’ results show that in 2006 all diseases are significant and have large odds ratio (OR) and consequently those individuals suffering from any of these diseases are more likely to report poor health. In 2011 the same pattern follows with the exception of allergies, obesity, high cholesterol and hypertension, albeit they are not statistically significant. Drinking habits had a positive effect on SRH in 2006 and 2011, whereas smoking is considered as unrelated to SRH. The likelihood of reporting poor health in 2006 is added as a variable in with the logistic regression of 2011 and is not, in either the ENSE data or the ESCA data, significant. Furthermore, neither is it significant when controlling by age, gender, employment status or education.

**Conclusions:**

The results of our analysis show that the financial crisis did not alter the likelihood of reporting poor health in 2011. Therefore, there are no differences between our perceived health in either 2006 or in 2011.

## Background

The introduction of the Euro (1999) in Spain led to a period of an economic boom based on the stock exchange market, which peaked at 125 % of the GDP in 2007, and a housing boom, which saw the construction sector go from 7.5 to 10 % of the GDP [1]. With the global financial crisis in 2008, the Spanish financial system collapsed as a result of its dependence on the construction sector and the lack of a growth model based on competitiveness. In 2010, 20 % of the population was unemployed and the external deficit had reached 10 % of the GDP. This debt continued to increase, reaching 90 % of the GDP in 2012 [2]. Thus, Spain is one of the countries that have most suffered from the global financial crisis. Catalonia, the region that accounts for 16 % of Spain’s population and almost a twenty per cent of its economy, has been defined as “another country” [3] since, apart from reasons of national identity, the financial crisis has not affected it as badly as other Spanish regions [2, 4].

One of the key results from the Spanish National Health Survey (ENSE) 2011–2012 is that 75.3 % of the population considered their health as being good or very good [5]. This is not only 5.3 points higher than that recorded in 2006, but it is also the highest percentage since the survey began. Interestingly, despite the financial recession and budget cuts that Spain has endured since 2008, it would seem that in fact people self-rate their health as much better than prior to the severe economic downturn. In this light two questions beg to be asked: (i) is the increase of the self-perceived health significant and, (ii) how can this improvement in subjective health be explained? Furthermore, the survey has also revealed that chronic pathologies such as arterial hypertension, high cholesterol, obesity and diabetes, in reality have actually continued to rise (for instance, high cholesterol in the population has increased from 8.2 to 16.4 % since 2003). Thus we sought to address whether the factors that affect self-rated health (SRH) changed or not during the financial crisis in Spain.

Self-rated health it is also known as self-assessed health or self-perceived health and is the subjective report of one’s health status reflecting “perceived” or “subjective” health. According to the World Health Organization (WHO), individuals rate their current status on a four or five point scale ranging from very good to very bad. Furthermore, all direct evidence of the health status of individuals is known as “actual” or “objective” health. Some health-related factors, such as life stress and/or life habits, are associated with objective health [6].

There is evidence that SRH is a strong predictor of morbidity and mortality [7–9]. There is also research that estimates the factors that influence SRH, provides objective measures and analyses the relationship between said factors [6, 10–12].

There is enough evidence to demonstrate the impact of an economic downturn on health [13, 14] and there are a number of studies testing how these factors affect SRH and how objective measures may change during a crisis. Åhs and Westerling find that the differences in SRH between the unemployed and the employed are greater when unemployment levels are high and that during periods of recession a greater number of unemployed groups are afflicted with poor health than when unemployment is low [15]. Bambra and Eikemo conclude that in every country in Europe, the unemployed report higher rates of poor health than those in employment [16]. The negative relationship between health and unemployment is consistent across Europe, but to what extent does this depend on the welfare state regime? On the other hand, Pawel and Worach-Kardas state that unemployment does not always have a detrimental effect on a person’s health and they find that unemployment does not significantly contribute to self-rated health status [17].

Some research in the United States suggests that during an economic downturn the health of the population actually improves [18]. Recently, Malat and Timberlake have shown that at the beginning of a period of high unemployment SRH averages are lowered. Yet, as unemployment rapidly increases, average health improves [19]. Also, there is a further line of thought which states that higher unemployment rates are associated with lower population mortality and higher unemployment serves to improve health [20].

In the ambit of the dire financial recession experienced in Greece, Zavras et al. put forward the idea that the likelihood of reporting poor SRH is higher in times of economic crisis and they also found some associations between SRH and the economic crisis [21]. Along these same lines, Kyriopoulos et al. argue that high-income earners seem to have better health and turn the spotlight away from themselves and onto the impact the economic crisis has on the health status of the middle and upper classes [22]. Vandoros et al. conclude that SRH has worsened as a result of the recent financial crisis [23].

In Spain, Regidor et al. attempted to identify any changes in health indicators during the financial crisis and concluded that Spanish health actually improved at a rate equal to or higher than what had occurred in the years prior to the recession [24].

Our objective is to examine whether the likelihood of reporting poor health has changed at all during the global financial crisis. The study is focused on estimating the differences between SRH, and other self-perceived measures of health, among groups both before and during the financial crisis in Spain.

## Methods

### Data setting

This study is based on the data we compared from two cross-sections of the Spanish National Health Survey, namely 2006 (i.e. prior to the economic downturn) and 2011, (in the middle of the economic crisis) [5]. We also used data from the Catalan Health Survey (ESCA), which is a survey that is similar to that of the ENSE but is carried out by Catalan government’s Department of Health [25]. Again, data before the economic crisis (ESCA-2006) was used and compared to the data amassed after the recession had begun (ESCA-2011). Even if ESCA and ENSE are rather similar, both data sets are interesting to use because of the economic differences between Spain and the region of Catalonia (4).

In 1987 the MSSSI (Ministry of Health, Social Services and Equity) implemented the, now periodic, ENSE study. Nowadays, the ENSE is conducted jointly between the MSSSI and the INE (National Statistics Institute). It is held every five years, alternating every two-and-a-half years with the European Health Survey, of which both surveys share some standardised variables. The ENSE aims to measure the characteristics and the distribution of morbidity in the Spanish population. Furthermore, it analyses the traits and distribution of behaviours and habits related to health and it also identifies the population’s use of medical services. All of these patterns relate to personal, demographic and territorial variables.

The ENSE survey consists of three questionnaires: one for households, another for adults and a third for minors (aged 0 to 15). A stratified tri-stage sample type is used. The first-stage unit is the census tract and the second-stage unit is the main family residence. One adult (aged 16 or over) is selected from each home to fill out the Adult’s Questionnaire and, should there be any minors, one is selected to fill out the Minor’s Questionnaire. A sample is uniformly assigned and in proportion to the size of the community. ENSE-2006 was conducted from June 2006 to June 2007. The sample of approximately 31,300 households distributed among 2236 census sections was selected, of which 29,478 of the collection were adults (direct interview) and 1822 minors (interviewed in the presence of their mother, father or guardian). The participation rate in this survey was 96 %. ENSE-2011 ran over a twelve month period from July 2011 to June 2012. The sample consisted of 21,508 households made up of 21,007 adults and 5495 children. Hence, 26,502 interviews were carried out. In this case, the participation rate was 71.08 %.

ESCA-2006 was completed between December 2005 and July 2006. There were 18,126 people interviewed, 15,926 of them were adults and 2200 were minors (aged 14 or younger). The response rate was 85.3 %. ESCA-2011 included some changes. The survey is now uninterrupted so that ESCA-2011 falls within the ESCA-2010–14 plan. The study is conducted in two stages with a total of 4828 people being interviewed, of whom 3901 were adults and 927 were minors (14 or younger).

### Statistical methods

Initially, we conducted four random effects logistic regressions which, in turn, obtained four models (ENSE 2006; ENSE 2011; ESCA 2006; ESCA 2011).

The dependent variable for the ENSE survey was the responses to the question, ‘How would you define your health status over the past 12 months?’ ‘Very good, good, fair, bad or very bad’. In the case of the ESCA survey, the question was, ‘How would you rate your general health?’ ‘Excellent, very good, good, fair or bad’. In order to have the same dependent variable for both surveys, we created a dependent dichotomous variable and regrouped the answers into good SRH (0), which includes excellent, very good and good, and poor SRH (1), which includes fair, bad and very bad.

We used several demographic, socio-economic and disease-related factors to evaluate influences on SRH. Confounding variables were gender (female, male) and age in years (15–35, 36–45, 46–55, 56–65, 66–75, 76+). An ordinal variable was education, which is grouped onto a four ordinal scale (no education/qualification, primary education, secondary education and tertiary education). Employment status was considered a nominal variable (employed, pensioner, student, housewife, short-term unemployment (-1 year) and long-term unemployment (+1 year)) as well as marital status (single, married, widowed, separated and divorced). Only for the ENSE survey did we also include a region variable in order to identify all the autonomous communities for a later comparison.

In the case of explanatory variables, we used some health measures as binary variables (no, yes) such as hypertension, diabetes, high cholesterol, varicose veins in the legs, upper back pain, lower back pain, chronic allergies, anxiety and depression, and migraine or frequent headaches. Note, headaches along with all the other illnesses taken into account were also subjective health measures (i.e. Have you had frequent headaches in the last 12 months?). Days of hospitalisation per year were used in ENSE and ESCA 2006 but in ESCA 2011 we used the number of hospitalisations per year instead (both categories were used as discrete variables). With lifestyle habits, we used smoking as a nominal variable (no, yes, in the past) and alcohol used as a binary variable (no, yes). We used the Body Mass Index (BMI) as an ordinal variable (underweight to normal, overweight, obese), and finally, the mental health variable is described on a scale of 0 (good mental health) to 12 (poor mental health) and calculated by each survey. However, with ESCA 2011 we used risk of poor mental health as a binomial (no, yes), which was also calculated by the survey. All these variables are self-rated measures so none of them can be considered objective measures.

We specified random effects logistic regressions. In mixed models terminology, we allowed (some of the) coefficients to be random effects [26], i.e. to be different for the various levels we considered. Thus, we allowed the intercept to be different for each individual, capturing specific individual characteristics not already included in the model (i.e. unobserved individual heterogeneity). In this case, we assumed that random effects were identical and independent of Gaussian random variables with constant variance (R-INLA project. Random walk model of order 1 (RW1)). (See more in http://www.math.ntnu.no/inla/r-inla.org/doc/latent/rw1.pdf).

As both the ENSE and the ESCA are cross-sections, respondents to either of the 2006 surveys are not the same respondents as those who participated in 2011. Therefore, the differences in the probability of declaring fair, poor or very poor health between 2006 and 2011, if any, could simply be as a consequence of the change in the composition of the sample. To make sure results were comparable we used exact matching. That is to say, we attempted to ensure that the ‘control’ (respondent of the ENSE or ESCA in 2006) matched each ‘case’ (respondent of the ENSE or ESCA in 2011) and had exactly the same values for the variables used for matching. In particular, the variables used for matching the same explanatory variables were entered into (random effects) logistic regressions for 2006.

Once the individuals were matched, we estimated two random effects logistic regressions for 2011 (i.e. one for the ENSE and one for the ESCA) including as a variable of interest the probability of declaring fair, poor or very poor health, (obtained as a result of the estimation of logistic regressions for 2006), and as control variables all the explanatory variables used in the logistic regressions for 2011.

Given the complexity of our model, we preferred to perform inferences using a Bayesian framework. This approach is considered the most suitable for accounting model uncertainty, both in the parameters and in the specification of the models. Furthermore, only under the Bayesian approach is it possible to model extra variability (not captured by the binomial link), with relatively sparse data in some cases. Finally, within the Bayesian approach, specifying a hierarchical structure on the (observable) data and (unobservable) parameters, which are all considered as random quantities, is straightforward. In particular, we followed the Integrated Nested Laplace Approximation (INLA) approach [27], within a (pure) Bayesian framework. All analyses have been made with the free software R (version 3.0.2) [28], available through the INLA library (R-INLA Project. See more in http://www.r-inla.org/home) [27].

## Results

Tables 1 and 2 (see tables online) show the descriptors of the samples used from the ENSE and the ESCA surveys, respectively. The first two columns (2006 and 2011, respectively) in each table depict the distribution of each and every one of the variables analysed, along with the percentage of those who declared their self-reported heath status as being poor. In the ENSE case (see Table 1) overall the descriptors show a slightly lower percentage of people with poor self-reported health in 2011, (compared to 2006), in all the variables minus in Aragón and the Canary Islands or in anxiety and depression where the 2011 percentages are slightly higher. In very general terms, the ESCA (see Table 2) illustrates that the percentage of those who identified themselves as having poor health dropped slightly from 2006 to 2011. However, this was the opposite case for those in the 15–35 year old age bracket, for those with chronic upper or lower back pain, or suffering from migraines. Neither was it true for those identified as having no education/qualification, secondary education or tertiary education, nor among those who were single or separated, as all of these groups showed higher percentages in 2011.

**Table 1:** Descriptive statistics for SRH in relation to demographic and socio-economic variables in ENSE survey

	% population in 2006 (*N* = 29,478)	% population in 2011 (*N* = 21,007)	% with poor SRH 2006	% with poor SRH 2011
Reported good SRH^a^	62.10	67.90		
Gender: Male	38.80	45.90	32.08	25.91
Female	61.20	54.10	43.80	37.32
Age: From 15 to 35 y.o.^b^	22.90	22.90	19.33	11.33
From 36 to 45 y.o.	19.90	18.70	26.01	18.58
From 46 to 55 y.o.	16.50	16.70	36.66	28.14
From 56 to 65 y.o.	14.50	14.90	50.26	39.82
From 66 to 75 y.o.	14.00	12.90	58.15	48.56
≥75 y.o	12.20	13.80	67.10	65.92
Regions: Catalonia	8.10	10.80	39.82	30.26
Andalusia	8.20	11.90	39.90	32.79
Aragon	9.30	4.10	34.94	36.00
Asturias	3.30	3.90	42.66	37.15
Balearic Islands	5.80	3.50	30.97	30.03
Canary Islands	3.80	5.10	38.48	38.98
Cantabria	5.90	3.60	34.43	32.04
Castile- Leon	4.50	6.20	35.91	31.56
Castile- La Mancha	3.70	4.90	39.05	31.28
Region of Valencia	5.70	8.10	42.07	31.16
Extremadura	3.10	4.10	44.28	33.76
Galicia	11.50	6.00	52.75	41.11
Madrid	6.90	9.20	34.10	25.26
Murcia	6.70	3.80	44.39	35.90
Navarre	5.60	3.70	34.53	25.91
Basque Country	3.70	5.60	32.77	30.30
La Rioja	2.40	3.40	30.96	28.51
Ceuta and Melilla	1.80	2.10	39.21	29.02
Hypertension	25.81	25.66	60.43	55.31
Diabetes	7.61	8.84	70.45	66.40
High cholesterol level	19.10	21.64	56.99	53.23
Varicose veins in the legs	15.91	14.25	60.58	56.61
Arthrosis, arthritis or rheumatism	26.82	23.89	71.82	68.12
Upper back pain	23.48	18.48	66.00	62.44
Lower back pain	23.94	21.77	65.60	59.93
Chronic allergies	11.89	12.32	44.67	38.45
Anxiety, depression	17.54	9.15	72.22	74.45
Migraine or frequent headaches	13.28	10.23	60.65	55.33
Days of hospitalisation	0.76	0.24		
Smoking				
No	52.40	54.70	43.84	34.88
Yes	26.60	25.30	31.32	25.28
In the past	21.00	19.90	37.86	32.96
Alcohol	52.30	49.30	32.14	23.08
BMI^c^				
Underweight to normal	46.00	45.20	30.26	22.54
Overweight	37.50	37.20	38.94	31.33
Obese	16.50	17.50	51.73	45.08
Mental health (mean)	1.68	1.59		
Education				
No education	14.40	14.60	66.97	62.20
Primary	46.60	44.60	43.86	35.53
Secondary	18.20	19.90	26.06	20.24
Tertiary	20.90	20.90	21.51	14.90
Employment status				
Employed	45.80	40.40	25.05	17.27
Pensioner	28.50	25.80	61.35	54.03
Student	3.30	5.90	12.07	6.94
Housewife	16.00	15.10	47.18	44.08
Short-term unemployed	5.00	6.40	32.03	18.92
Long-term unemployed	1.40	6.40	45.25	31.84
Marital status				
Single	24.10	28.20	26.86	19.68
Married	57.40	52.30	39.20	31.66
Widowed	12.90	13.10	62.49	59.21
Separated	3.10	2.40	38.44	34.51
Divorced	2.50	4.00	41.62	34.84

^a^self-rated health

^b^years old

^c^Body Mass Index

**Table 2:** Descriptive statistics for SRH in relation to demographic and socio-economic variables in ESCA survey

	% population in 2006 (*N* = 15,926)	% population in 2011 (*N* = 3901)	% with poor SRH 2006	% with poor SRH 2011
Reported good SRH^a^	74.30	76.30		
Gender: Male	49.50	50.10	20.54	19.68
Female	50.50	49.90	30.74	27.71
Age: From 15 to 35 y.o.^b^	33.40	32.80	7.49	8.29
From 36 to 45 y.o.	17.90	17.90	14.84	12.05
From 46 to 55 y.o.	14.90	14.40	26.05	21.39
From 56 to 65 y.o.	12.50	13.20	37.89	33.33
From 66 to 75 y.o.	10.60	9.40	50.09	43.84
≥ 75 y.o	10.60	12.50	61.85	58.11
Hypertension	21.10	25.71	49.96	44.47
Diabetes	6.28	8.02	64.70	60.06
High cholesterol level	15.36	21.10	46.47	40.83
Varicose veins in the legs	19.86	19.35	46.82	44.77
Arthrosis, arthritis or rheumatism	24.37	27.20	59.31	51.27
Upper back pain	28.01	25.07	46.90	48.26
Lower back pain	30.13	29.61	46.19	48.66
Chronic allergies	15.70	14.84	31.51	31.09
Anxiety, depression	17.83	20.51	55.99	53.38
Migraine or frequent headaches	18.87	19.41	42.96	43.86
Days of hospitalisation	0.7			
Number hospitalisations		0.13		
Smoking				
No	52.30	51.60	27.64	24.52
Yes	28.20	28.70	17.42	17.05
In the past	19.40	19.80	25.77	23.89
Alcohol	71.90	66.60	19.74	17.62
BMI^c^				
Underweight to normal	50.20	50.60	18.70	14.29
Overweight	36.40	35.30	29.36	19.30
Obese	13.40	14.10	42.27	35.90
Mental health				
Mean	0.78			
Risk poor mental health		11.90		
Education				
No education/qualification	14.70	12	56.80	58.00
Primary	42.40	38.20	29.22	26.95
Secondary	20.30	21.90	13.71	15.83
Tertiary	22.60	27.90	9.54	10.65
Employment status				
Employed	58.80	51.70	13.13	11.58
Pensioner	17.30	18.90	50.57	46.28
Student	6.40	8.60	3.69	5.03
Housewife	13.10	10.20	44.60	42.71
Short-term unemployed	3.50	8.90	24.13	13.72
Long-term unemployed	0.90	1.80	36.36	26.15
Marital status				
Single	30.50	31.60	11.64	11.92
Married	57.10	55.10	28.39	25.10
Widowed	8.30	8.20	57.81	57.01
Separated	2.50	2.50	28.25	28.87
Divorced	1.70	2.50	26.77	26.26

^a^self-rated health

^b^years old

^c^Body Mass Index

Tables 3 and 4 show the results obtained from the initial estimations of the logistics models. In Table 3 (ENSE 2006) we can see how the different variables introduced into the model influence the likelihood of claiming to be in poor health. Having said this, this does not hold true for the variables of gender, for those who are overweight, for the short-term unemployed, or for all those (minus the widowed) in the marital status category, nor does it hold true for some of the autonomous communities. ENSE 2011 follows very similar lines as 2006 ENSE, i.e. some autonomous communities, gender, those who are overweight, the short-term unemployed or those married or widowed, did not significantly influence the probability of declaring one’s health status as poor (the 95 % credible interval did contain the zero). We must point out that in ENSE 2006 individuals (with any disease taken into account) tend to report a poorer self-rated health [odds ratio (OR)≥1]. Particularly, the probability of rating poor health increases when an individual suffers from diabetes, arthritis or depression [OR≥2]. As for BMI, being overweight is not statistically significant, whereas being obese (BMI > 30 kg/m2) does have a negative impact on reported health [OR = 1.29]. Mental health disorders also worsen self-reported health [OR = 1.27] as does days of hospitalization [OR = 1.15]. In terms of habits, currently smoking [OR = 1.18] or being an ex-smoker [OR = 1.14] increases the probability of rating one’s health as poor, whereas alcohol usage increases the probability of reporting good health [OR = 0.77]. With demographic and socio-economic variables, age indicates that older individuals have a higher probability of rating their health as poor or very poor. Surprisingly, gender is not statistically significant. In terms of education, the higher the education level, the lower the OR observed were, so individuals with tertiary studies are less likely to report poor health [OR = 0.46]. Employment status indicates that pensioners [OR = 1.60] and housewives [OR = 1.13] have a higher probability than the employed of rating their health as poor. Although the short-term unemployed are not statistically significant, the long-term unemployed are more likely to report poorer health than those who are employed [OR = 1.48]. Students were not statistically significant at all. Finally, marital status showed that only widowed or separated individuals reported better health than singles [OR≤1] and being married or divorced was not statistically significant. However, the data from the 2011 Spanish National Health Survey did highlight some differences. All the diseases examined along with BMI, mental health, days of hospitalization, smoking, alcohol habits and education follow the same pattern, whereas age and its impact on self-reported health changed. In 2011 OR are greater than in 2006. So the likelihood of reporting poor health as you get older is much greater. Again, gender seems to be unrelated to self-reported health (*p* > 0.05). In terms of employment status, it seems that differences between being employed and/or any other situation have been reduced. For example, pensioners [OR = 1.30] and the long-term unemployed [OR = 1.30] still rate their health as poor but not as bad as they did in 2006. Students are still better off than employed individuals [OR = 0.61], but the differences are smaller than in 2006. The only group whose status had increased its impact was housewives [OR = 1.21]. Short-term unemployment remains unrelated to SRH. Finally, once again those widowed report better health than those who are single [OR = 0.68] and now also better than those who are married [OR = 0.87]. Being divorced or separated is not statistically significant.

**Table 3:** Logistic regression of poor SRH^a^ on demographic and socio-economic variables in ENSE survey

	2006		2011	
	OR (95 % CI)		OR (95 % CI)	
Gender: (Male)				
Female	1.04	(0.96, 1.12)	0.96	(0.87, 1.05)
Age (From 15 to 35 years old)				
From 36 to 45 y.o.^b^	1.13	(1.02, 1.25)	1.29	(1.11, 1.49)
From 46 to 55 y.o.	1.39	(1.25, 1.56)	1.54	(1.33, 1.78)
From 56 to 65 y.o.	1.50	(1.32, 1.70)	1.76	(1.49, 2.08)
From 66 to 75 y.o.	1.38	(1.18, 1.62)	1.92	(1.57, 2.35)
≥ 75 y.o	2.00	(1.69, 2.36)	3.66	(2.96, 4.52)
Regions (Catalonia)				
Andalusia	0.72	(0.62, 0.83)	0.93	(0.79, 1.09)
Aragon	0.70	(0.61, 0.81)	1.21	(0.99, 1.49)
Asturias	0.73	(0.60, 0.89)	0.90	(0.72, 1.12)
Balearic Islands	0.50	(0.42, 0.59)	0.78	(0.62, 0.99)
Canary Islands	0.69	(0.57, 0.83)	1.16	(0.95, 1.41)
Cantabria	0.88	(0.75, 1.04)	0.74	(0.59, 0.91)
Castile- Leon	0.69	(0.58, 0.82)	0.90	(0.75, 1.08)
Castile- La Mancha	0.70	(0.58, 0.84)	0.77	(0.62, 0.94)
Region of Valencia	1.01	(0.86, 1.18)	0.97	(0.82, 1.16)
Extremadura	0.84	(0.69, 1.03)	0.79	(0.64, 0.99)
Galicia	1.22	(1.07, 1.40)	1.22	(1.01, 1.47)
Madrid	0.63	(0.54, 0.74)	0.81	(0.68, 0.96)
Murcia	1.06	(0.91, 1.24)	1.02	(0.82, 1.27)
Navarre	0.63	(0.54, 0.75)	0.72	(0.57, 0.91)
Basque Country	0.73	(0.60, 0.88)	0.90	(0.74, 1.09)
La Rioja	0.83	(0.67, 1.04)	0.88	(0.70, 1.11)
Ceuta and Melilla	0.79	(0.62, 1.02)	1.50	(1.15, 1.96)
Hypertension	1.38	(1.28, 1.49)	1.39	(1.27, 1.52)
Diabetes	2.04	(1.81, 2.30)	1.85	(1.63, 2.12)
Cholesterol	1.25	(1.15, 1.35)	1.44	(1.32, 1.58)
Varicous veins	1.24	(1.14, 1.35)	1.23	(1.11, 1.37)
Arthrosis, arthritis or rheumatism	2.44	(2.26, 2.63)	2.37	(2.16, 2.61)
Upper back pain	1.67	(1.54, 1.81)	1.60	(1.44, 1.78)
Lower back pain	1.93	(1.78, 2.09)	1.76	(1.60, 1.94)
Allergy	1.26	(1.14, 1.38)	1.32	(1.17, 1.47)
Anxiety, depression	2.11	(1.94, 2.30)	1.86	(1.63, 2.14)
Migraine	1.54	(1.40, 1.69)	1.57	(1.39, 1.78)
Days of hospitalisation	1.15	(1.14, 1.17)	1.00	(1.00, 1.01)
Smoking (No)				
Yes	1.18	(1.09, 1.28)	1.24	(1.12, 1.37)
In the past	1.14	(1.05, 1.23)	1.16	(1.05, 1.29)
Alcohol	0.77	(0.72, 0.82)	0.71	(0.66, 0.76)
BMI^c^ (Underweight to normal)				
Overweight	1.03	(0.95, 1.11)	1.00	(0.92, 1.10)
Obese	1.29	(1.18, 1.42)	1.26	(1.13, 1.42)
Mental health	1.26	(1.25, 1.28)	1.24	(1.22, 1.26)
Education (No education/qualification)				
Primary	0.72	(0.65, 0.79)	0.72	(0.65, 0.81)
Secondary	0.58	(0.51, 0.65)	0.53	(0.46, 0.62)
Tertiary	0.46	(0.41, 0.52)	0.46	(0.40, 0.54)
Employment status (Employed)				
Pensioner	1.60	(1.43, 1.80)	1.30	(1.12, 1.50)
Student	0.57	(0.45, 0.71)	0.61	(0.47, 0.79)
Housewife	1.13	(1.02, 1.25)	1.21	(1.05, 1.38)
Short-term unemployed	1.13	(0.98, 1.30)	0.93	(0.78, 1.12)
Long-term unemployed	1.48	(1.16, 1.88)	1.30	(1.11, 1.52)
Marital status (single)				
Married	0.98	(0.90, 1.07)	0.87	(0.78, 0.97)
Widowed	0.76	(0.67, 0.87)	0.68	(0.59, 0.80)
Separated	0.83	(0.69, 1.00)	0.82	(0.64, 1.06)
Divorced	1.11	(0.90, 1.37)	1.06	(0.87, 1.29)
Mean	0.46		0.43	
DIC^d^	26929.17		18098.01	
EPO^e^	57.29		57.07	
Random effects-heterogeneity	5.4E–05	(7.86E–04, 1.49E–05)	5.4E–05	(7.96E–04, 1.50E–05)

^a^self-rated health

^b^years old

^c^for Body Mass Index

^d^deviance information criterion

^e^effective number of parameters

**Table 4:** Logistic regression of poor SRH^a^ on demographic and socio-economic variables in ESCA survey

	2006		2011	
	OR (95 % CI)		OR (95 % CI)	
Gender (Male)				
Female	0.97	(0.87, 1.10)	0.76	(0.59, 0.97)
Age (From 15 to 35 years old)				
From 36 to 45 y.o.^b^	1.53	(1.28, 1.84)	0.99	(0.68, 1.45)
From 46 to 55 y.o.	2.14	(1.79, 2.57)	1.47	(1.00, 2.18)
From 56 to 65 y.o.	2.19	(1.78, 2.68)	1.40	(0.91, 2.15)
From 66 to 75 y.o.	2.70	(2.10, 3.46)	1.48	(0.85, 2.56)
≥ 75 y.o	4.12	(3.17, 5.35)	2.13	(0.92, 4.95)
Hypertension	1.42	(1.28, 1.59)	1.20	(0.96, 1.51)
Diabetes	2.33	(1.96, 2.76)	2.34	(1.71, 3.21)
Cholesterol	1.21	(1.07, 1.34)	1.15	(0.91, 1.44)
Varicous veins	1.34	(1.20, 1.49)	1.31	(1.04, 1.66)
Arthrosis, arthritis or rheumatism	1.98	(1.77, 2.22)	1.80	(1.44, 2.25)
Upper back pain	1.53	(1.37, 1.71)	1.36	(1.08, 1.73)
Lower back pain	1.74	(1.56, 1.93)	2.62	(2.10, 3.27)
Allergy	1.32	(1.17, 1.50)	1.15	(0.88, 1.51)
Anxiety, depression	1.78	(1.58, 2.00)	2.32	(1.84, 2.92)
Migraine	1.39	(1.24, 1.56)	2.11	(1.66, 2.66)
Days of hospitalisation	1.05	(1.03, 1.06)	1.52	(1.25, 1.89)
Smoking (No)				
Yes	1.23	(1.08, 1.39)	1.02	(0.78, 1.33)
In the past	1.11	(0.98, 1.27)	0.84	(0.64, 1.10)
Alcohol				
Alcohol (No drinking)				
Moderate drinking	0.75	(0.68, 0.83)	0.76	(0.61, 0.94)
Risk drinking	0.74	(0.56, 0.96)	0.66	(0.38, 1.13)
BMI^c^ (Underweight to normal)				
Overweight	1.13	(1.02, 1.26)	0.90	(0.70, 1.17)
Obese	1.30	(1.13, 1.49)	1.49	(1.09, 2.04)
Mental health	1.29	(1.25, 1.32)		
Mental health (normal)				
Risk poor mental health			2.45	(1.84, 3.27)
Education (No education/qualification)				
Primary	0.85	(0.75, 0.97)	0.61	(0.46, 0.82)
Secondary	0.63	(0.53, 0.74)	0.53	(0.37, 0.76)
Tertiary	0.47	(0.39, 0.56)	0.38	(0.27, 0.55)
Employment status (Employed)				
Pensioner	1.60	(1.33, 1.92)	2.12	(1.40, 3.21)
Student	0.56	(0.38, 0.82)	0.70	(0.34, 1.37)
Housewife	1.30	(1.10, 1.53)	1.49	(1.01, 2.19)
Short-term unemployed	1.59	(1.24, 2.03)	1.06	(0.70, 1.58)
Long-term unemployed	1.40	(0.88, 2.22)	1.42	(0.70, 2.78)
Marital status (single)				
Married	1.09	(0.95, 1.26)	0.96	(0.71, 1.30)
Widowed	0.86	(0.70, 1.06)	0.99	(0.64, 1.54)
Separated	1.19	(0.88, 1.61)	0.96	(0.49, 1.84)
Divorced	0.92	(0.63, 1.32)	0.82	(0.43, 1.52)
Mean	0.37		0.35	
DIC^d^	11861.07		inf	
EPO	40.98		inf	
Random effects- heterogeneity	5.37E–05	(7.90E–04, 1.49E–05)	5.38E–05	(7.90E–04, 1.49E–05)

^a^self-rated health

^b^years old

^c^for Body Mass Index

^d^deviance information criterion

The results of ESCA 2006 (Table 4) were in line with ENSE 2006. Almost all of the variables introduced into the model (with the exception of marital status, long-term unemployment,being an ex-smoker or a female) affected the likelihood of reporting poor health. However, this would vary considerably in 2011 when many of the variables became statistically insignificant. These variables included age (albeit only in the 46–55 year age group), hypertension, high cholesterol, allergies, current smoker, alcohol use, being overweight, being a student and the short-term unemployed. We should highlight the fact that being female now increases the likelihood of reporting good health [OR = 0.76]. In terms of education, having higher levels of studies increases the chance of reporting good health when compared to 2006. In 2011 there are smaller OR and this shows that in 2011 studies were more important for reporting good health than in 2006.

Lastly, Table 5 contains (for ENSE 2011 and ESCA 2011, respectively), the results from the logistics models using as the explanatory variable (from 2006) the likelihood of perceiving oneself as being in poor health. In both cases the variable was not statistically significant (the 95 % credible interval did contain the zero), i.e. the likelihood of declaring poor health in 2006 had no bearing on whether poor health was declared or not in 2011. The tables show the same results for all the categories that were analysed (gender, age, education levels and employment status).

**Table 5:** Probability of reporting poor SRH in 2006 in Logistic Regression 2011

***ENSE data*** SRH^a^	0.09	(−0.26, 0.44)
For groups lineal approach		
Gender [Male]	−0.21	(−1.08, 0.66)
Gender [Female]	0.30	(−0.10, 0.71)
Age [From 15 to 35 years old]	0.98	(−2.24, 4.18)
Age [From 36 to 45 y.o.^b^]	0.34	(−1.82, 2.50)
Age [From 46 to 55 y.o.]	−1.60	(−3.25, 0.04)
Age [From 56 to 65 y.o.]	−0.27	(−1.25, 0.71)
Age [From 66 to 75 y.o.]	0.60	(−0.47, 1.67)
Age [≥ 76 y.o.]	0.47	(−0.07, 1.02)
Education [No education]	0.30	(−0.31, 0.88)
Education [Primary]	−0.26	(−0.76, 0.26)
Education [Secondary]	1.78	(−0.85, 4.39)
Education [Tertiary]	−1.08	(−4.31, 2.14)
Employment status [Employed]	0.14	(−0.22, 0.50)
Employment status [Unemployed]	−1.81	(−3.92, 0.24)
***ESCA data*** SRH^a^	−0.07	(−0.57, 0.43)
For groups lineal approach		
Gender [Male]	−0.20	(−1.21, 0.79)
Gender [Female]	0.06	(−0.54, 0.66)
Age [From 15 to 35 years old]	0.96	(−0.37, 2.21)
Age [From 36 to 45 y.o.^b^]	0.04	(−1.57, 1.57)
Age [From 46 to 55 y.o.]	−0.14	(−1.76, 1.33)
Age [From 56 to 65 y.o.]	−1.15	(−2.65, 0.31)
Age [From 66 to 75 y.o.]	1.35	(−0.03, 2.75)
Education [No education]	0.42	(−0.66, 1.51)
Education [Primary]	−0.23	(−1.05, 0.58)
Education [Secondary]	0.25	(−0.98, 1.44)
Education [Tertiary]	−0.16	(−1.48, 1.08)
Employment status [Employed]	−0.07	(−0.59, 0.44)
Employment status [Unemployed]	0.71	(−1.52, 2.86)

^a^self-rated health

^b^years old; the 95 % credible interval did not contain the zero (statistically significant)

## Discussion

This is the first study to analyse the increase in SRH in the 2006 and 2011 surveys carried out in Spain. Our results show that the probability of reporting poor health in 2006 is, despite the grave economic recession the country is experiencing, not significantly different in 2011 and so we have been able to conclude that people rate their health equally in both of these years.

In general, SRH improves in all the countries which have been affected by the global financial crisis such as the USA, Japan and Europe; albeit with the exception of Greece. However, these conclusions are based on cross-sectional data so the individuals involved are not the same and it has been proven that this can bias the results [29, 30].

In order to control any cross-sectional problems that could change the results, we matched individuals from 2006 to 2011 by using the same health factors and demographic characteristics. Since the probability in 2006 of reporting poor health is not significant in the logistic regression for 2011 (both ENSE and ESCA), we can conclude that the individuals rate their health equally for both years; even in the economic downturn.

Interestingly the same conclusions were drawn during the Baltic States transition from 1994–1999 [31]. Other studies which also concern the current recession, report that the health of the population does not have to decline during a financial crisis [17, 32, 33]. Furthermore, in concordance with our results, Urbanos-Garrido and López-Valcárcel [34], using both the Spanish Health Survey for the years 2006 and 2011–2012 and, above all, a matching technique, find that the change in the percentage of (self-declared) poor health after the crisis, for the total population (never employed, unemployed and employed), although negative, was not statistically significant. In contrast, Regidor *et al.* [24], although they did not use the same statistical method (matching in particular) find that poor self-reported health showed statistically significant downward trends during the recession.

Our results are not consistent with the findings in the studies coming from Greece which conclude that the economic crisis does in fact have a negative impact on SRH [21–23]. However, only Vandoros *et al.* [23] use an experimental method to control for observational effects and, therefore, their results are directly comparable with ours. Having said this, the short-term consequences of the crisis in Greece, for both SRH and suicide indicators, have proved worse than in Spain. As Stuckler et al. pointed out in their work, Greece has experienced the highest increase in suicides between 2007 and 2008 (17 %) [35], whereas, according to Lopez Bernal et al., Spain has suffered an increase in the suicide rate 8 % above the trend [36]. On the other hand, the long-term consequences for both countries are expected to be severe and irreversible due to the increase in poverty, unemployment and social exclusion that both countries are experiencing.

Therefore, most of the studies, including our own, find that the perception of poor health did not increase as a consequence of the crisis or the cuts in health care budgets. On the one hand, it has been argued that this fact could be explained, at least in Europe, by the effect of unemployment insurance. Ferrarini et al. [37] point out that unemployment insurance mitigated adverse health effects both on an individual and a country-level during the financial crisis [16, 17]. In Spain, however, perhaps because unemployment levels double those of the European average, unemployment in general and long-term unemployment in particular, has had a significant negative impact on self-assessed health. This is true in both our own case as well as in that of Urbanos-Garrido and López-Valcárcel [34], in spite of both studies’ entire samples not registering any statistically significant impact. On the other hand, and according to López-Casasnovas [38], so far cuts in health care budgets in Spain do not appear to have resulted in lower health care quality.

In general, people rate their health by taking into account a certain reference group or situation. Thus, people compare themselves to others (friends, relatives) and their current situation (in terms of health, job and income) [10, 12, 17, 19, 29]. We believe that today the reference group or situation has changed (decreased) and as a consequence people now overrate their health because they know that as a result of the financial recession the situation around them is much more difficult and complicated. This would seem to be why they rate their health as better than prior to the recession, when in fact they should rate their health equally; as our results show.

### Limitations

We acknowledge that this study has its limitations. First, we analysed SRH as a dichotomous (good and poor SRH) dependent variable instead of a categorical variable (excellent, very good, good, regular, bad and very bad SRH) so that differences between the levels of answers cannot be perceived. Second, all the measures of health we used are based on self-reported health and so increase the probability of recall bias. Also, even if SRH is generally accepted as being valid, reliable and predictive of mortality [7–9], it might not have the same meaning to everyone because it is subject to an individual’s perceptions and expectations. Finally, there are further measures, which have not included in our analysis, but which can also affect our SRH.

## Conclusions

The economic crisis in Spain is associated with increased income inequality and this leads to some possible effects on health. Most people tend to think that the economic crisis has had a negative impact on health because of an increase in certain chronic diseases such as hypertension, high cholesterol, obesity and diabetes, and a negative impact on the SRH. Despite this, our results show that the majority of Spanish citizens describe their health as good or very good, so we can conclude that people value their health equally for both years; even in the economic downturn.

Therefore, our findings confirm that the crisis does in fact have an impact on health. In other words, if the crisis had no impact on SRH, our findings would have shown a decrease in the likelihood of reporting poor health. So, governments should take this study into account because the trend has changed and SRH is no longer increasing. This is indeed significant and decision-makers must take this into consideration and design policies that protect the health of all the different groups that make up the population.

In a situation such as that which currently exists in our country, where the recession has lasted a considerable amount of time, governments have been forced to make cuts to public budgets in order to reduce the budget deficit, and this affects the most vulnerable population groups such as the long-term unemployed. The authorities should pay more attention to these groups and continue researching ways to avoid this. In this sense, the future challenge for policy, in view of the current economic scenario agents, is to prioritize cost-effective evaluation of public policies to improve equity, efficiency and the quality of health care.

## Key points

- This is the first study to analyse the increase in SRH in the 2006 and 2011 surveys carried out in Spain.
- The probability of reporting poor health in 2006 is not significantly different in 2011.
- Decision-makers must take into consideration that SRH actually do not improve, in contrast to the last National Health Survey’s conclusion (ENSE).
- Cross-sectional problems can be controlled with exact matching and propensity score-matching.

## References

[1] Weisbrot M. Los problemas de España provienen de las políticas de la zona del euro. Center for Economic and Policy Research. Washington DC: USA; 2011.

[2] Spanish National Statistics Institute (INE). Basic data. http://www.ine.es. Accessed 10 Oct 2013.

[3] Buck T (2014). Catalonia: another country. The financial times.

[4] Sala-i-Martin X (2014). És l’hora dels adéus? Rosa dels Vents.

[5] Ministry of Health, Social Services and Equity: National Health Survey in Spain 2006 and 2011. Householder’s questionnaire and adult’s questionnaire: http://www.msssi.gob.es/estadEstudios/estadisticas/encuestaNacional/encuesta2006.htm. Accessed 15 Sept 2013. http://www.msssi.gob.es/estadEstudios/estadisticas/encuestaNacional/encuesta2011.htm. Accessed 15 Sept 2013.

[6] Wu S, Wang R, Zhao Y (2013). The relationship between self-rated health and objective health status: A population-based study. BMC Public Health.

[7] Jylhä M (2009). What is self-rated health and why does it predict mortality? Towards a unified conceptual model. Soc Sci Med.

[8] Idler EL, Benyamini Y (1997). Self-rated health and mortality: a review of twenty-seven community studies. J Health Soc Behav.

[9] Kaplan GA, Camacho T (1983). Perceived Health and Mortality: a nine-year follow-up of the human Population Laboratory Cohort. Am J Epidemiol.

[10] Gumà J, Cámara AD (2014). ¿Informa la salud autopercibida sobre las condiciones objetivas de salud? Algunas conclusiones a partir del análisis demográfico de microdatos de la Encuesta Nacional de Salud. Estadística Española.

[11] Gupta S, Adam E, McDade T. Objective versus Subjective Measures of Health: Systematic differences, determinants and biases. (Preliminary version 2010).

[12] Baker M, Stabile M, Deri C (2004). What do self-reported, objective, measures of health measure?. J Hum Resour.

[13] Marmot M, Bell R (2009). How will the financial crisis affect health?. BMJ.

[14] Musgrove P (1987). The economic crisis and its impact on health and health care in Latin America and the Caribbean. Int J Health Serv.

[15] Åhs A, Westerling R (2006). Self-rated health in relation to employment status during periods of high and of low levels of unemployment. Eur J Public Health.

[16] Bambra C, Eikemo TA (2009). Welfare state regimes, unemployment and health: A comparative study of the relationship between unemployment and self-reported health in 23 European countries. J Epidemiol Community Health.

[17] Pawel S, Worach-Kardas H (2014). Living with long-term unemployment- effects in health and quality of life. J Health Sci.

[18] Catalano R (2009). Health, medical care, and economic crisis. N Engl J Med.

[19] Malat J, Timberlake JM (2013). County-level unemployment change and trends in SRH. Sociol Focus.

[20] Tapia Granados JA, Diez-Roux AV (2009). Life and death during the Great Depression. Proc Natl Acad Sci U S A.

[21] Zavras D, Tsiantou V, Pavi E, Mylona K, Kyriopoulos J (2012). Impact of economic crisis and other demographic and socio-economic factors on self-rated health in Greece. Eur J Public Health.

[22] Kyriopoulos II, Zavras D, Pavi E, Kyriopoulos J (2012). Socioeconomic Inequalities Concerning the Self-Rated Health Status in Greece: A Comparative Analysis of Post-Crisis Effects. Value Health J.

[23] Vandoros S, Hessel P, Leone T, Avendano M (2013). Have health trends worsened in Greece as a result of the financial crisis? A quasi-experimental approach. Eur J Public Health.

[24] Regidor E, Barrio G, Bravo MJ, De la Fuente L (2013). Has health in Spain been declining since the economic crisis?. J Epidemiol Community Health.

[25] General Direction of Regulation, Planning and Health Resources. Department of Health, Government of Catalonia. Catalan Health Survey in Catalonia 2006 and 2010-2014. Methodology in detail. Government of Catalonia. 2012. http://salutweb.gencat.cat/ca/el_departament/estadistiques_sanitaries/enquestes/enquesta_salut_catalunya. Accessed 10 Oct 2013.

[26] Pinheiro JC, Bates D (2000). Mixed-effects Models in S and S-Plus.

[27] Rue H, Martino S, Chopin N (2009). Approximate Bayesian inference for latent Gaussian models by using integrated nested Laplace approximations (with discussion). J R Stat Soc Ser B.

[28] R Core Team. R (2013). A language and environment for statistical computing.

[29] Groot W (2003). Scale of reference bias and the evolution of health. Eur J Health Econ.

[30] Orfila F, Ferrer M, Lamarca R, Alonso J (2000). Evolution of self-rated health status in the elderly: Cross-sectional vs. longitudinal estimates. J Clin Epidemiol.

[31] Monden CWS (2005). Changing social variations in self-assessed health in times of transition? The Baltic States 1994-1999. Eur J Pub Health.

[32] Dávila Quintana CD, González L-VB (2009). The economic crisis and health. Gac Sanit.

[33] Ruhm CJ (2005). Healthy living in hard times. J Health Econ.

[34] Urbanos-Garrido RM, López-Valcárcel BG (2015). The influence of the economic crisis on the association between unemployment and health: an empirical analysis for Spain. Eur J Health Econ.

[35] Stuckler D, Basu S, Suhrcke M (2011). Effects of the 2008 recession on health: a first look at European data. Lancet.

[36] López-Bernal JA, Gasparrini A, Artundo C (2013). The effect of the late 2000s financial crisis on suicides in Spain: an interrupted time-series analysis. Eur J Public Health.

[37] Ferrarini T, Nelson K, Sjöberg O (2014). Unemployment insurance and deteriorating self-rated health in 23 European countries. J Epidemiol Community Health.

[38] López-Casasnovas G. La crisis económica española y sus consecuencias sobre el gasto social. Informe SESPAS 2014 Gac Sanit. 2014;28 Supl 1:18–23. 10.1016/j.gaceta.2014.02.020 24863990

